# Patient-experience during delivery in public health facilities in Uttar Pradesh, India

**DOI:** 10.1093/heapol/czz067

**Published:** 2019-08-16

**Authors:** Dominic Montagu, Amanda Landrian, Vishwajeet Kumar, Beth S Phillips, Shreya Singhal, Shambhavi Mishra, Shambhavi Singh, Sun Yu Cotter, Vinay Pratap Singh, Fnu Kajal, May Sudhinaraset

**Affiliations:** 1 School of Medicine, Department of Epidemiology and Biostatistics, University of California, San Francisco, Mission Hall 550 16th St. 3rd Floor, San Francisco, CA, USA; 2 Jonathan and Karin Fielding School of Public Health, University of California, 650 Charles E. Young Dr. South, Los Angeles, CA, USA; 3 Community Empowerment Lab, Lucknow, Uttar Pradesh, India; 4 Department of Statistics, University of Lucknow, Lucknow; 5 National Health Mission, Lucknow, Uttar Pradesh, India

**Keywords:** Maternal health, quality, global health, person-centred-care

## Abstract

In India, most women now delivery in hospitals or other facilities, however, maternal and neonatal mortality remains stubbornly high. Studies have shown that mistreatment causes delays in care-seeking, early discharge and poor adherence to post-delivery guidance. This study seeks to understand the variation of women’s experiences in different levels of government facilities. This information can help to guide improvement planning. We surveyed 2018 women who gave birth in a representative set of 40 government facilities from across Uttar Pradesh (UP) state in northern India. Women were asked about their experiences of care, using an established scale for person-centred care. We asked questions specific to treatment and clinical care, including whether tests such as blood pressure, contraction timing, newborn heartbeat or vaginal exams were conducted, and whether medical assessments for mothers or newborns were done prior to discharge. Women delivering in hospitals reported less attentive care than women in lower-level facilities, and were less trusting of their providers. After controlling for a range of demographic attributes, we found that better access, higher clinical quality, and lower facility-level, were all significantly predictive of patient-centred care. In UP, lower-level facilities are more accessible, women have greater trust for the providers and women report being better treated than in hospitals. For the vast majority of women who will have a safe and uncomplicated delivery, our findings suggest that the best option would be to invest in improvements mid-level facilities, with access to effective and efficient emergency referral and transportation systems should they be needed.

**Key Messages**

- In Uttar Pradesh, India, patient experience, clinical attentiveness and counselling, and patients’ trust of doctors is worst in the higher-level hospitals where clinical infrastructure is best.
- These findings may explain why women delay going to hospitals for maternity care, discharge themselves early, and why high levels of maternal mortality and morbidity persist.

## Background

In India, over 90% of women become mothers in their lifetime (Hulton *et al.*, 2007). Giving birth carries risks, but these risks are mitigated by early presentation for care, attendance by a skilled birth attendant, access to clinical services and the availability of a functioning health referral system to reach emergency services when necessary (Gabrysch *et al.*, 2012). All of these risk mitigators are most likely present in a healthcare facility—a clinic or hospital—and so the priority across India, and globally, for the past 50 years has been to move labour and delivery from homes to facilities whenever possible (Campbell and Graham, 2006).

This shift from home delivery to facility-based deliveries has happened in India exceptionally quickly over the past decade, spurred by a well-funded and well-publicized national conditional-cash-transfer programme named Janani Suraksha Yojana (JSY). JSY has led to a rapid and widespread increase in facility-based deliveries, from 18% in 2008, to >80% 10 years later (Anand *et al.*, 2016; Montagu *et al.*, 2017; Salve *et al.*, 2017). While it has risen quite a bit, the rate is lower in the northern state of Uttar Pradesh (UP) than in the rest of India overall: in UP, only 68% of women give birth in a hospital or clinic, and the state has a maternal mortality rate of 285 deaths per 100 000 live births, 1.7 times higher than that of India as a whole (*NFHS-4 2015/6*, 2016). UP is the most populous state in India, underscoring the importance of better understanding drivers of poor outcomes there.

Past studies have shown that women delay seeking care from facilities because they fear mistreatment or poor quality of care from healthcare providers and staff (Kruk *et al.*, 2009, 2010). Recent studies of both public and private hospitals and clinics in UP have shown that quality across many domains—safety, patient-centredness, equity, accessibility, efficiency, effectiveness, to list just a few—is often low (Sharma *et al.*, 2017). Quality in government facilities is of particular concern, especially in UP as the majority of deliveries take place in government facilities (National Family Health Survey, 2014).

In collaboration with the National Health Mission of UP, we examined women’s experiences in these facilities. Research from other countries has shown that higher-level facilities—referral and specialty hospitals—provide better clinical care but worse patient-centred care than lower-level facilities (Sjetne *et al.*, 2007; McFarland *et al.*, 2017). This study seeks to expand our understanding of these issues in India, and to describe the patient experience for women who deliver in government health facilities in UP.

Both Community Health Centre/ First Referral Unit (CHC/FRU) and District Women’s Hospitals (DWHs) should be staffed with at least one Ob/Gyn and be capable of providing round-the-clock emergency obstetrics and newborn care (EmONC), although in practice in UP this is not always the case at the CHC/FRU level (Chokshi *et al.*, 2016; Sharma *et al.*, 2017). Little is known about patient experience across different levels of care. While studies have shown better quality of care in higher-level facilities, this has not been found in UP (Kruk *et al.*, 2009; Sharma *et al.*, 2017). Following national guidelines, confirmed by observation, higher-level facilities in UP are better equipped than lower-level Primary Health Centres (PHCs) and CHC (Ministry of Health & Family Welfare, 2016). Internationally, better infrastructure is associated, poorly but positively, with better clinical care (Leslie *et al.*, 2017). It is possible that in UP, as elsewhere, patient experience may suffer in higher-level facilities which document high client volumes, staff shortages and overburdened health systems (Young *et al.*, 2000). We hypothesize that while clinical quality may be higher in district hospitals (high-level facilities), they will also report the lowest levels of the patient experience.

We examine surveys across UP as part of a larger study looking at facility characteristics associated with better and worse maternity patient care experiences. Past studies in UP and elsewhere in India have identified women’s preferences for perceived higher clinical competence when seeking a facility, and also the importance of treatment and respect (Bhattacharyya *et al.*, 2016, 2018; Sharma *et al.*, 2017). Given variations in these attributes by facility type and size, we are particularly interested in the quality of care across and how it may differ by facility-level as well during points of time in the process of accessing a facility, during labour and delivery, and post-partum.

## Methods

### Context

**Figure 1:**
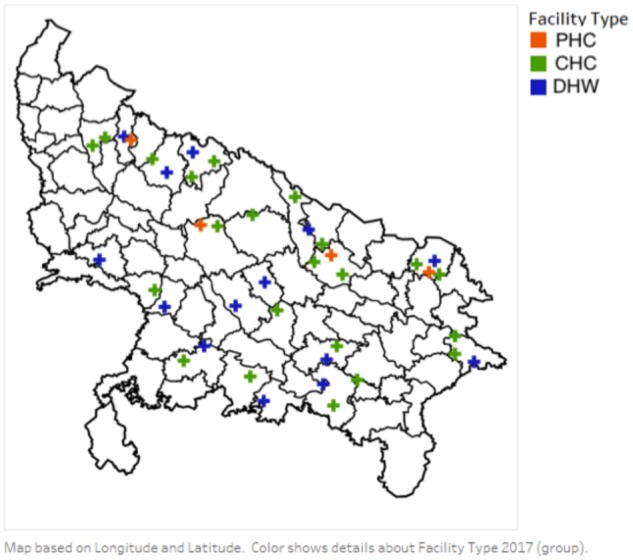
Map of study sites in Uttar Pradesh, India (*n* = 40).

The sample frame for our study was drawn from nearly 750 public hospitals and clinics across UP, a state in northern India. Ten facilities from each of the state’s 75 districts were selected to include all large and high-volume facilities. We first identified all CHC/FRUs and all hospitals in each district, and all other ‘high-volume’ facilities, reporting delivery volumes >200/month during the quarter prior to selection; the criteria to be defined as high volume according to past studies in the state (Sharma *et al.*, 2017). All remaining facilities in the district were randomly selected to make a total of 10 per state. These 750 facilities were surveyed by the National Health Mission for basic data on infrastructure, service volumes and staffing. In total, 727 responded and were used as the frame for our sample. We followed Nesbit et al.’s (2013) methodology to sum maternal health service indicators to an overall ‘maternal health service performance’ scores for each facility and then ranked facilities according to this score. Next, we stratified by geography and facility type and based on this purposefully selected a geographically and quality representative set of 40 facilities from 20 districts for in-depth study (see Figure 1) (Palinkas *et al.*, 2015).

Our research included patients from facilities at all four levels of the UP system: 4 PHC, 10 CHC, 12 CHC/FRU and 14 DWHs. PHCs are the lowest level of in-patient care infrastructure, often situated in rural communities and are designed to serve populations of 20 000–30 000. PHCs were not initially intended to provide delivery services; however, this changed and in^ 2013^ a national NMCH+A policy initiated support for an expansion of trained birth attendants at PCHs [A Strategic Approach to Reproductive, Maternal, Newborn, Child and Adolescent Health (RMNCH+A) in India, 2013]. From PHCs, delivery patients are referred to CHCs, if complications arise, then from CHCs to CHC/FRUs, and if needed from there to DWHs (Chokshi *et al.*, 2016).

### Delivery patient surveys

The delivery patient survey was administered to approximately 50 patients per study site (*n* = 2018). The survey was conducted from August 2017 through October 2017 and contained questions related to patient’s demographics, antenatal care, labour and delivery services and outcomes, and her self-reported person-centred maternity care (PCMC) experience. Questions asked about access (why this facility, how did you get here, who decided), care upon arrival (reception, politeness, examinations), during labour and delivery (clinical care, information given, timeliness, management of any complications), as well as social support and costs. The patient’s experience with PCMC was assessed using a validated PCMC scale for India which includes subscales for dignity and respect, communication and autonomy, and supportive care (Afulani *et al*., 2018). Eligible women (adults 18 years of age and over whom had delivered within 48 h preceding survey at a study facility) were identified by data collectors based on information from facility staff, and subsequently recruited to participate in the survey. Recruitment and consent occurred at the post-natal ward; after confirming eligibility, women were told about the study goals and invited to participate. In most facilities, all eligible women were approached during daytime hours (∼7 am to 7 pm). Security concerns prevented researchers from staying in or travelling to facilities after dark. Women who delivered at night were approached in the morning if still in the facility. In high-volume facilities, women were approached randomly according to presentation after the completion of another interview. Respondents could choose their preference to continue with the interview in a private space at the facility or at their bed. Nearly all women preferred to be interviewed at the post-natal ward at their patient bed. All interviews were conducted in Hindi and took 45 min to complete on average. All women were interviewed only with a family member or other support person present as per the study protocols. About 2040 women were consented, 21 identified as ineligible after consenting, and 1 subject withdrew for other reasons. Within each facility, interviews continued until 50 were completed.

Interviewers were hired and trained by the local partner research institute. All had with prior health research experience in Uttar Pradesh. They participated in a week-long training on survey techniques, the delivery and provider surveys, participant recruitment methods and public health research ethics. From August to November 2017, two male interviewers conducted all the providers surveys (*n* = 250) while 20 female interviewers administered the delivery surveys (*n* = 2018). After receiving additional training on the qualitative interview guides and qualitative research practice, a subset of six survey interviewers conducted the qualitative interviews (*n* = 50) from November 2017 to February 2018.

### Measures

We divided facilities into ‘lower-level’ and ‘higher-level’ based on their nominal capacity to provide basic emergency obstetric care or emergency obstetric care (EmOC) according to government guidelines (Bhattacharyya *et al.*, 2016, 2018; Sharma *et al.*, 2017). In UP, some facilities function at a different level from their formal classification, but we have retained the government ranking to assure objectivity in our categorization. All PHCs and CHCs were classified as ‘lower-level’, while FRU-CHCs and DWHs were classified as ‘higher-level’.

Respondent’s wealth quintiles were calculated using the EquityTool methodology of household asset ownership categorization, based on asset ownership derived state-level quintiles created from the National Family Household Survey (National Family Health Survey, 2014). A binary variable was then created to capture whether the respondent’s wealth fell within the bottom 40% (i.e. the lowest two wealth quintiles).

While it is clear that patient-centred measures of care do not specifically assess clinical quality, they are inextricably linked. Our survey included several questions on the process of seeking and receiving care which reflect aspects of clinical management but are not part of the Afulani PCMC Scale (Afulani *et al*., 2018). Accessibility of services, attentiveness of care at the time of facility arrival, during labour and delivery, and after delivery, along with the receipt of counselling before discharge, are all important measures of service management that focus on clinical staff practices rather than patient respect or patient-centred care experiences as incorporated into the PCMC Scale. Patients were considered to have experienced low transportation barriers in accessing care if they reported that their transportation to the health facility was short in time, easy to access and affordable.

### Clinical care

In addition to transportation barriers (Accessing Care), we assessed the clinical quality of care at three points in time: upon arrival at a facility (Care Upon Arrival); during labour and delivery (Care During Delivery); and post-partum (Care After Delivery). Care upon arrival to the facility was assessed by asking women if a health provider checked their blood pressure, checked their pulse, timed their contractions, listened to the baby’s heartbeat and performed a vaginal examination. Cut offs for quality were determined based on the distribution of health-checks received across the sample, creating a criteria to differentiate better and less-good care, providing differentiation of patient recall of clinical care rather than a validated measure of objective clinical quality. Women who received at least three of these health checks were considered to have received good attention upon arrival to the facility. Women were considered to have received good care during labour and delivery if they received at least three of the same health checks and if they endorsed that a clinical staff member (doctor, nurse and/or midwife) attended the delivery, that the baby was put on their chest or abdomen immediately after delivery, and that a health provider checked on the health of them and their baby immediately after delivery. To assess the quality of care received after delivery, women were asked if a health provider checked their blood pressure, checked their pulse, examined their abdomen, examined their perineum, checked their pad for bleeding, examined the baby, checked to ensure breastfeeding was going well and asked if urine and stool were being easily passed. Women who reported receiving at least four health checks, and who also reported being visited by a health provider after being moved to the general ward, were considered to have received good care after delivery. Women were also asked if they received counselling on breastfeeding at any point after delivery but before being discharged, which was included as an additional variable for assessing post-partum care.

### Analyses

Data were analysed using descriptive, bivariate and multivariate statistics, without additional cluster analysis. Pearson’s chi-square tests were used to examine differences in patient demographic characteristics and patient-reported quality of care indicators by facility-level. Bivariate logistic regression was used to examine facility-level as a predictor of patient-reported quality of care indicators. Multiple linear regression was used to examine the patient-reported quality of care indicators and facility-level as predictors of PCMC scores. All analyses were completed in StataSE version 15.1 (StataCorp, 2017).

Ethical clearance for this research was provided by the ethics review boards of the authors’ institutions.

## Results

### Patience demographic characteristics

Most women who give birth in UP do so in a facility, and of those, most deliver in a government facility. In our study, the religious and caste divisions were closely representative of the state of UP, with Hindu and Muslims making up 83% and 17% of the sample (compared with 79.7% and 19.3% for the state), and Scheduled or Other Backward Castes making up 28% and 55% of the sample (compared with 20.5% and 40% for the state) (National Family Health Survey, 2014.).

Patient demographic characteristics, stratified by facility-level of care, are shown in Table 1. Most women surveyed were between 20 and 29 years old, married, unemployed outside the home, and having their first or second child. Women in higher-level facilities were more likely to be having their first child than those in lower-level facilities (*P* < 0.001), with 43% of deliveries in District Hospitals to nulliparous women compared between 29% and 32% in all other facilities. Women in PHCs were more likely (65.8%) to have only primary school education or less than women in CHCs (49.6%), CHC/FRUs (48.4%) or DWHs (37.4%) (*P* < 0.001). Women who gave birth in hospitals were wealthier than those who delivered in lower-level facilities.

**Table 1:** Patient demographic characteristics by facility-level, *N* = 2018.

Characteristic	Level of care				*P*-value^a^
	PHC (*n* = 202)	CHC (*n* = 504)	FRU-CHC (*n* = 609)	DWH (*n* = 703)	
Age (years), %					<0.001
15–19	44.1	47.0	44.5	55.9	
20–29	44.6	45.6	44.0	38.7	
30–48	11.4	7.3	11.5	5.4	
Marital status, %					0.70
Married	99.5	99.8	99.8	99.7	
Separated	0.5	0.0	0.2	0.1	
Widowed	0.0	0.2	0.0	0.1	
Education, %					<0.001
Primary or less	65.8	49.6	48.4	37.4	
Post-primary/Vocational/Secondary	30.7	37.5	41.2	46.4	
College or higher	3.5	12.9	10.3	16.2	
Employment status, %					0.16
Unemployed	96.0	95.4	92.8	94.6	
Employed	4.0	4.6	7.2	5.4	
Occupation, %					<0.001
Agricultural labor	3.0	2.8	3.0	0.3	
Casual labor	0.5	0.8	3.1	2.0	
Salaried worker	0.0	0.6	0.5	1.7	
Self-employed in petty trade	0.5	0.4	0.7	1.4	
Unemployed/Homemaker	96.0	95.4	92.8	94.6	
Residence, %					<0.001
Urban	100.00	96.2	92.9	67.1	
Rural	0.0	3.8	7.1	32.9	
Religion, %					0.02
Hindu	81.7	79.2	86.0	83.5	
Muslim	18.3	20.8	14.0	16.2	
Other	0.0	0.0	0.0	0.3	
Bottom 40% in wealth, %					<0.001
Yes	52.0	42.5	45.7	30.0	
No	48/0	57.5	54.4	70.0	
Caste, %					<0.001
General	18.8	15.3	10.7	21.6	
Other backward class	55.9	56.9	56.8	52.1	
Scheduled caste or tribe	25.3	27.8	32.5	26.3	
Parity, %					<0.001
1	29.2	29.6	32.0	43.1	
2	27.7	32.5	29.6	29.7	
3	18.8	21.2	20.0	17.9	
4 or more	24.3	16.7	18.4	9.3	

Percentages may not add to 100 due to rounding.

a *P*-values shown are for Pearson’s chi-square tests.

The same women were also asked about services and treatment received as part of exit-interviews (Table 2). Counselling for breastfeeding was reported more than two-thirds of the time in all facility types, but less in hospitals than in PHCs, CHCs or FRU-CHCs. For all of the tracked quality-indicators, patients reported worse care in hospitals than in other settings (*P* < 0.001). Women delivering in DWHs were more likely to report significant challenges in transportation to their place of delivery than women in lower-level facilities. Better post-partum care was reported by women who delivered in PHCs than in the three higher levels of the facility (*P* < 0.001).

**Table 2:** Patient-reported quality of care indicators by facility-level, *N* = 2018.

Characteristic	PHC (*n* = 202)	CHC (*n* = 504)	FRU-CHC (*n* = 609)	DWH (*n* = 703)	*P*-value^a^
Experienced low transportation barriers, %					<0.001
Yes	38.8	48.9	39.9	17.1	
No	61.2	51.1	60.1	82.9	
Received good attention upon arrival to facility, %					<0.001
Yes	37.6	30.4	38.1	12.5	
No	62.4	69.6	61.9	87.5	
Received good care during labour and delivery, %					<0.001
Yes	33.2	12.3	26.8	5.8	
No	66.8	87.7	73.2	94.2	
Received good care after labour and delivery, %					<0.001
Yes	25.3	5.2	10.2	1.6	
No	74.8	94.8	89.8	98.4	
Received counselling on breastfeeding, %					<0.001
Yes	80.5	78.9	78.6	67.7	
No	19.5	21.1	21.4	32.3	

Percentages may not add to 100 due to rounding.

a *P*-values shown are for Pearson’s chi-square tests.

### Women’s experiences of care, by facility-level

**Figure 2:**
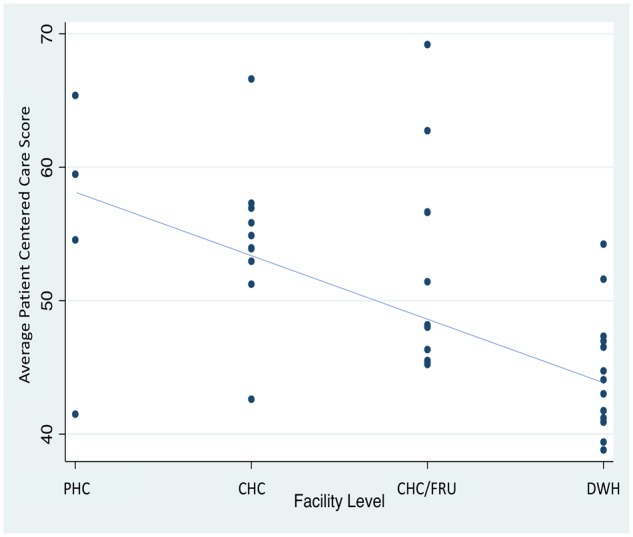
Average patient-centred care score, by facility-level with linear trendline.

Figure 2 shows our application of a validated scale for person-centred maternal care to all 2018 patient exit surveys. Bivariate analysis of facility type against facility-average PCMC score shows that higher-level facilities provide a worse patient experience for women. Higher-level facilities scored, on average, 15% lower on the PCMC scale than lower-level facilities.

Bivariate logistic regression analysis confirmed this result and showed that client assessment of the attentiveness of care across the continuum of care decrease as the level of care in which clients deliver increases (Table 3).

**Table 3:** Bivariate logistic regression examining differences in clinical care received by facility-level

	Received good attention upon arrival to facility OR (95% CL)	Received good care during labour and delivery OR (95% CL)	Received good care after delivery OR (95% CL)
Facility-level (referent: PHC)			
CHC	0.72 (0.51–1.02)	0.28 (0.19–0.42)***	0.16 (0.10–0.27)***
FRU-CHC	1.02 (0.73–1.42)	0.74 (0.52–1.04)	0.34 (0.22–0.51)***
DWH	0.23 (0.17–0.34)***	0.12 (0.08–0.19)***	0.05 (0.02–0.09)***

* *P* < 0.05;

** *P* < 0.01;

*** *P* < 0.001.

**Figure 3:**
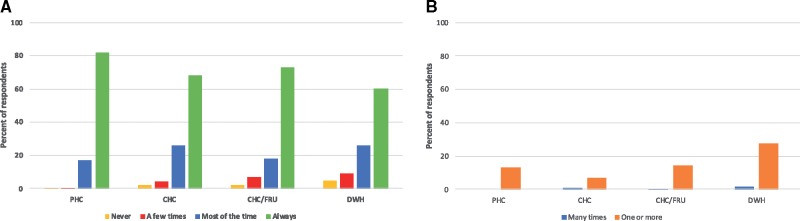
Patient reporting on (A) trusting their provider and (B) experiences with verbal abuse, by facility type.

Women delivering in hospitals are also less likely to report trusting their provider, and women in all higher-level facilities are more likely to report experiencing verbal abuse during their delivery than women in lower-level facilities (Figure 3A and B).

### Clinical care and patient-centred care

We conducted multiple linear regression on six models with aspects of patient-reported clinical care and facility-level as predictors of PCMC score (Table 4). After controlling for age, education, caste, urban/rural residence, wealth and parity, we found ease of access to care, quality of clinical care at arrival, during delivery and after delivery, and facility-level, respectively, to be significantly predictive of PCMC score at a significance of less than *P* < 0.001 (Models 1–5). In Model 6, which examines the continuum of care by adding all variables to a single model, we found that quality of clinical care upon arrival, during delivery and after delivery was positively predictive of higher PCMC scores at a significance of *P* < 0.001. We also found higher facility-level to be negatively predictive of PCMC score (*P* < 0.001). This was true after adjusting for age, education, caste, urban/rural residence, wealth and parity. Ease of access to care was no longer significantly predictive of PCMC scores in the full model.

**Table 4:** Multiple linear regression examining clinical care as a predictor of patient-centred maternity care scores

Variables	PCMC score coeff. (95% CI)
Model 1: Accessing care	
Experienced low transportation barriers in accessing care	3.21 (2.23, 4.21)***
Constant	50.64 (49.03, 52.25)***
Model 2: Facility-level	
PHC	Ref
CHC	−1.27 (−2.90, 0.36)
CHC-FRU	−3.75 (−5.34, 2.15)***
DWH	−11.59 (−13.24, −9.95)***
Constant	52.89 (50.77, 55.01)***
Model 3: Care upon arrival	
Received good attention upon arrival to facility	7.40 (6.39, 8.40)***
Constant	49.50 (47.95, 51.05)***
Model 4: Care during delivery	
Received good care during labour and delivery	10.08 (8.89, 11.27)***
Constant	49.44 (47.91, 50.96)***
Model 5: Care after delivery	
Received good care after labour and delivery	6.67 (4.89, 8.44)***
Received counselling on breastfeeding	4.22 (3.16, 5.29)***
Constant	47.50 (45.72, 49.28)***
Model 6: Examining continuum of maternal care at labour and delivery	
Experienced low transportation barriers	0.90 (−0.00, 1.80)
Facility-level	
PHC	Ref
CHC	0.82 (−0.77, 2.40)
FRU-CHC	−2.74 (−4.27, 1.21)***
DWH	−7.79 (−9.43, −6.16)***
Received good attention upon arival to facility	2.58 (1.51, 3.66)***
Received good care during labour and delivery	6.76 (5.46, 8.05)***
Received good care after labour and delivery	1.84 (0.15, 3.53)*
Received counselling on breastfeeding	2.34 (1.37, 3.30)***
Constant	47.55 (45.36, 49.73)***

All models control for patient’s age, education, caste, place of residence, wealth and parity.

* *P* < 0.05;

** *P* < 0.01;

*** *P* < 0.001.

## Discussion

The limited capacity to manage complications in lower-level PHCs and CHCs is intentional—these facilities are not required by the Federal Ministry of Health or the state’s Department of Health to provide blood transfusions, caesarean sections and other emergency obstetric services (EmOC): higher-level facilities are better equipped, better staffed and intended to be able to provide more comprehensive care (National Health Systems Resource Center, 2017). Referral systems should transport those who need emergency care to the appropriate FRU-CHC or DWH. Transportation networks in UP including innovative public–private ambulance services are, as in many parts of India, a visible success (Singh *et al.*, 2016, 2018).

Transportation alone cannot make up for overall health system challenges, however. Our study has found that aspects of care involving attentiveness and attendance to clients are not aligned with this scale of better quality in higher-level centres. In multiple models, we found that clinical experiences—attention, care and counselling—were strongly and positively correlated with higher levels of patient-centred care, regardless of facility type. This confirms findings from other studies, and may indicate, among other things, the likelihood of a recall bias associated with good clinical outcomes (having a healthy baby).

Our study shows the limits of this association, in finding that higher-level facilities provider a worse experience for women, scoring lower on person-centred care even after adjusting for clinical care. One hypothesis is that this could be the result of expectation bias: we know poorer and less educated women are more likely to deliver in lower-level sites. Their greater satisfaction may be the result, not of better person-centred care, but of lower expectations which are therefore easier to meet by facility staff. Our analysis shows this to be unlikely. While there are differences in the demographic characteristics of women across facility types, with wealthier, more urban and lower parity women more likely to deliver in higher-level facilities and poorer, rural, multiparous women more likely to deliver in lower-level facilities, the relationship between clinical care and patient-centred care remained strong in all of our models after adjusting for these factors.

Other underlying factors, such as relative crowding, provider stress or workload and lack of personal connections due to volume, may all contribute to the differential experiences in facilities of different levels.

Our study has a number of limitations. We use cross-sectional data, which allow us to understand associations but not causal links. Our frame is based on self-reported data from facilities collected by the National Health Mission, and so may be unreliable which would affect our selection of facilities. Past studies have highlighted recall biases specifically for delivery experiences. Interviews close to the time of delivery may lead to a positive bias as women focus on the success of delivery and their newborn son (Gibbins and Thomson, 2001; Sando *et al.*, 2017). Interviews conducted at the facilities, as ours were, are also open to bias as women may be unwilling to be critical of the providers who are nearby, or the facility in which they still reside (Freedman and Kruk, 2014; Abuya *et al.*, 2018).

## Conclusions

Our study found that higher-level facilities in UP provide worse patient-centred treatment than lower-level facilities. There is a linear relationship between facility-level and worse patient-centred care and this same relationship can be seen in patient reporting on care and attention throughout the continuum of care, from arrival and admission, to labour and delivery, and post-delivery care.

Influential thinking about how best to assure quality maternity services has highlighted client preferences for clinically advanced tertiary facilities, and the efficiency gains possible by investing in a smaller number of well-used facilities rather than a large number of staffed clinics with few clients (Kruk *et al.*, 2018). This argument is based in large part upon documented experiences—preferences and positive outcomes—for maternal health ‘bypassing’ in East African (Kruk *et al.*, 2009). Our findings in UP imply that a similar strategy may not be appropriate for Northern India.

In UP, lower-level facilities are more accessible, women have greater trust for the providers and women report being better treated than in hospitals. For the vast majority of women who will have a safe and uncomplicated delivery, our findings suggest that the best option would be to deliver in a lower-level centre with access to effective and efficient emergency referral; or better yet to deliver in a mid-level facility where patient treatment is better and EmOC services already exist. Active quality improvement efforts in UP being led by the Technical Support Unit/iHAT in conjunction with the National Health Mission have expanded in recent years to simultaneously address lower and higher-level facilities, and the referral systems that link them (Sridharan *et al.*, 2017). At the same time, other recent intervention initiatives in UP have shown that client-experiences and services processes in lower and mid-level facilities can be improved (Semrau *et al.*, 2017; Phillips *et al.*, 2018).

In this context, our findings thus suggest that quality improvement efforts in India prioritize investments in CHCs and CHC/FRUs as a way to build on the comparatively better patient experience in these facilities and also assure clinical quality. Within hospitals and FRUs, attention is needed to address poor patient-centred care. Investments in management systems and staff in these facilities are likely to be more beneficial than the introduction of new equipment.

Person-centred care is a key component of overall maternal health quality. The experiences of women during labour and delivery influence their adherence to medical advice post-partum, their care seeking after discharge, and quite probably the attitudes of themselves and their family members and friends towards future delivery and ANC services. Good clinical services must be paired with good person-centred care, and balancing the attention to each aspect of care will be important for future quality improvement efforts in India.

*Ethical approval*: Ethical approvals were received from the UCSF Internal Review Board, and the CEL Institutional Ethics Committee.
