# Patient-experience during delivery in public health facilities in Uttar Pradesh, India

**DOI:** 10.1093/heapol/czz125

**Published:** 2019-11-12

**Authors:** Dominic Montagu, Amanda Landrian, Vishwajeet Kumar, Beth S Phillips, Shreya Singhal, Shambhavi Mishra, Shambhavi Singh, Sun Yu Cotter, Vinay Pratap Singh, Fnu Kajal, May Sudhinaraset

**Affiliations:** 1 School of Medicine, Department of Epidemiology and Biostatistics, University of California, San Francisco, Mission Hall 550 16th St. 3rd Floor, San Francisco, CA 94158, USA; 2 Jonathan and Karin Fielding School of Public Health, University of California, Los Angeles, 650 Charles E. Young Dr. South, Los Angeles, CA 90095, USA; 3 Community Empowerment Lab, Lucknow, Uttar Pradesh, India; 4 Department of Statistics, University of Lucknow, Lucknow; 5 National Health Mission, Lucknow, Uttar Pradesh, India

*Health Policy and Planning*, doi: 10.1093/heapol/czz067

This manuscript has been amended to make corrections to the spelling of three co-authors’ names, and one affiliation which changed after submission. The author apologises for any confusion caused.

